# Increased skeletal intermuscular fat is associated with reduced exercise capacity in cancer survivors: a cross-sectional study

**DOI:** 10.1186/s40959-019-0038-5

**Published:** 2019-05-03

**Authors:** Kerryn W. Reding, Peter Brubaker, Ralph D’Agostino, Dalane W. Kitzman, Barbara Nicklas, Dale Langford, Michael Grodesky, W. Gregory Hundley

**Affiliations:** 1 Department of Biobehavioral Nursing and Health Informatics, University of Washington School of Nursing, Seattle, WA USA; 2 Department of Health and Exercise Science, Wake Forest University, Winston-Salem, NC USA; 3 Department of Biostatistical Sciences, Wake Forest School of Medicine, Winston-Salem, NC USA; 4 Department of Internal Medicine (Section on Cardiology), Wake Forest School of Medicine, Medical Center Boulevard, Winston-Salem, NC 27157-1045 USA; 5 Gerontology and Geriatric Medicine, Wake Forest School of Medicine, Winston-Salem, NC USA; 6 Department of Anesthesiology and Pain Medicine, University of Washington School of Medicine, Seattle, WA USA; 7 Department of Psychosocial and Community Health, University of Washington School of Nursing, Seattle, WA USA; 8 Pauley Heart Center, Virginia Commonwealth University Health System, Richmond, VA USA; 9 Department of Cancer Prevention, Public Health Sciences, Fred Hutch Cancer Research Center, Seattle, WA USA

**Keywords:** Exercise intolerance, Muscle composition, Cancer survivors

## Abstract

**Background:**

Cancer survivors experience on average a 20% reduction in peak exercise capacity (VO_2 peak_) post-cancer treatment. Intermuscular fat (IMF) is a strong predictor of reduced exercise capacity in heart failure (HF) patients; however it is unknown whether increased IMF is related to reduced VO_2 peak_ in cancer survivors.

**Methods and results:**

Twenty eight individuals: 14 cancer survivors > 12-months post-cancer treatment and 14 individuals without cancer were matched on age, gender, and body mass index (BMI). Participants underwent magnetic resonance imaging (MRI) assessments of IMF within the paraspinal muscles, VO_2 peak_ and exercise-associated measures of left ventricular ejection fraction (LVEF). Blinded analyses were performed. Associations between the ratio of IMF to skeletal muscle (SM) were estimated using Pearson’s partial correlation coefficients. Individuals with cancer and non-cancer comparators were of similar age (54 ± 17 versus 54 ± 15 years; *p* = 1.0), gender (5 men and 9 women, both groups), and BMI (27 ± 4 versus 26 ± 4; *p* = 0.57). Peak VO_2_ was 22% lower in cancer survivors versus non-cancer comparators (26.9 vs 34.3 ml/kg/min; *p* = 0.005), and was correlated with IMF:SM in both cancer survivors and non-cancer individuals after accounting for exercise-associated LVEF, resting LVEF, BMI, other body fat depots, and cardiovascular disease (CVD) co-morbidities (*p* < 0.001 to 0.08 for all adjusted correlations).

**Conclusion:**

Among cancer survivors that previously received anthracyclines, increased intermuscular fat is associated with reduced VO_2 peak_ even after accounting for exercise-associated cardiac function. This suggests IMF is important in the development of exercise intolerance, an outcome experienced by a large number of cancer survivors.

## Introduction

Peak exercise capacity (VO_2 peak_) is reduced by an average 20% among cancer survivors who received potentially cardiotoxic treatment for their cancer [1]. According to the Fick equation, VO_2 peak_ is a function of cardiac output and the arterio-venous oxygen (a-VO_2_) difference, a measure indicating the ability of the periphery to extract oxygen from the circulating blood [2]. Given the association between anthracycline-based chemotherapy and reduced left ventricular ejection fraction (LVEF), research in cancer survivors has predominantly focused on the role of cardiac dysfunction in reduced exercise capacity as opposed to factors that may impact the a-VO2 difference.

However, in non-cancer populations reductions in the exercise-associated a-VO2 difference predicts reduced VO_2 peak_ [3]. Adipose tissue, particularly intermuscular fat (IMF), is metabolically active and competes with skeletal muscle for tissue perfusion and oxygen consumption [4, 5]. A study in heart failure patients supported this notion, showing that the ratio of intermuscular fat (IMF) to skeletal muscle (SM) predicted reductions in VO_2peak_ [5]. Moreover, emerging data shows that IMF accumulates during cancer treatment [5]. The finding that IMF increases over the same period during which exercise capacity is reduced led us to evaluate the relationship between the IMF:SM ratio in paraspinal skeletal muscles and VO_2 peak_ in cancer survivors.

## Methods

We enrolled 14 cancer survivors > 12-months after receipt of anthracycline-based chemotherapy, who were matched on age, gender, and body mass index (BMI) to 14 individuals without cancer serving as non-cancer comparators. This matching ensured that cancer survivors and non-cancer comparators were < 1 unit apart on BMI and age, and that the range of values was within 1 and 2 units, respectively. Results on these participants have been presented in an International forum with the Society for Cardiovascular Magnetic Resonance [6]. Cancer survivors were identified from the hematology and oncology clinics at the Comprehensive Cancer Center at Wake Forest Health Sciences in Winston-Salem, North Carolina. The cancer survivors and non-cancer comparators had no prior myocardial infarction, heart failure, or other prior cardiovascular event. The exclusion criteria for cancer survivors and non-cancer comparators were contraindication for magnetic resonance imaging (MRI), including a pacemaker, defibrillator, or other implanted electronic devices; inability to perform exercise treadmill testing; pregnancy; claustrophobia; acute illness or injury related to walking briskly or running on a treadmill; or incapability to provide informed consent. All participants provided informed consent. This study was approved by the Institutional Review Board of the Wake Forest University School of Medicine.

**Fig. 1:**
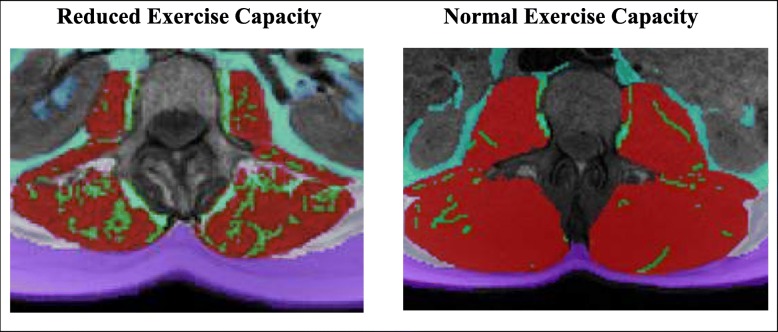
The intermuscular fat of paraspinal muscles, depicted from MRI images of increased IMF (green) relative to SM (red) in a cancer survivor with reduced exercise capacity (left) and a control participant with normal exercise capacity (right)

According to previously published techniques [7], all participants underwent abdominal MRI with determination of IMF and SM in the paraspinal muscles, and subcutaneous, intraperitoneal, and retroperitoneal fat. Images were acquired with an axial non-contrast T1 weighted MRI scan positioned at the level of the second lumber vertebra (L2) using a 5-mm thick slice, a 256 × 256 matrix, and a 180^°^ flip angle. TomoVision SliceOmatic version 5.0 was used to measure muscle and adipose tissue depots (Fig. 1). A MRI analyst manually separated muscle and fat using reproducible, previously described methods [5]. Briefly, the area of each adipose and muscle compartment was calculated as number of pixels multiplied by pixel surface area. IMF was defined as the adipose tissue visible by MRI within the boundary of the muscle fascia.

At the time of IMF acquisition, LVEF was measured from a series of cine white blood images (8 mm thick with 2 mm gap, temporal resolution 40 msec, field of view 36 cm, and 256 × 128 matrix) spanning the left ventricular (LV) base to apex acquired during 6 to 8-s of breath-holding. LV volumes were determined using a modified Simpson’s rule calculation, and the LVEF was determined by subtracting the LV end-systolic volume from the LV end-diastolic volume and dividing by the LV end-diastolic volume [7]. Images were analyzed by individuals blinded to all participant characteristics and other testing results.

After acquiring images to measure LV function at rest and paraspinal fat, each participant underwent a cardiopulmonary exercise treadmill stress test (CPET) measuring VO_2 peak_. For this, participants performed a treadmill test according to the Bruce protocol or modified Bruce protocol (depending on fitness level) to the point of maximal exercise as identified by trained staff who were blinded to their survivorship or non-cancer status. Immediately after attaining peak exercise, participants transferred back to the scanner and underwent acquisition of LV function within 50 s of exercise cessation, henceforth called exercise-associated LVEF.

Prior to MRI, each participant completed the Godin Leisure Time Physical Activity Questionnaire to ascertain self-reported physical activity [8]. This questionnaire measures the frequency of strenuous, moderate, and mild exercise performed during a typical 7 day period and is reported in times per week of exercise > 15 min in duration. Participants also completed the functional assessment of cancer therapy: fatigue (FACT–F) questionnaire, a 13-item questionnaire developed to assess fatigue in cancer patients over the last seven days [9]. Scores range from zero to 52 with a higher score indicating lower fatigue levels. A categorical variable indicating the number of CVD co-morbidities was created based on the presence of a) coronary artery disease, b) diabetes, and c) hypertension (using resting blood pressure and current use of antihypertensive medications).

Control and cancer patient groups were compared on baseline characteristics using 2-sample t-tests for continuous measures and Fisher’s exact tests for binary measures. Next, Pearson correlation coefficients were estimated to examine the correlation between peak VO_2_ measures and intermuscular fat to skeletal muscle ratio separately for control and cancer patients. These correlations were estimated unadjusted, and adjusted individually for resting LVEF, exercise-associated LVEF, BMI, subcutaneous fat, and intraperitoneal fat.

## Results

Five men and nine women were included in each group. Cancer survivors and controls were of similar age (54 ± 17 and 54 ± 15 years) and BMI (27 ± 4 and 26 ± 4), respectively. Eight and six of the cancer survivors had breast cancer and lymphoma, respectively, with an average cumulative received dose of 327 ± 139 mg/m^2^ in doxorubicin equivalents [10]. The proportion of participants with CVD co-morbidities was similar across the two groups, with six cancer survivors and four non-cancer comparators reporting 1+ CVD co-morbidity (*p* = 0.20). Cancer survivors and non-cancer comparators were similar with respect to self-reported fatigue (46 ± 6 and 48 ± 3, respectively; *p* = 0.28) and times per week of engagement in mild (3.5 ± 2.5 and 3.4 ± 3.0, respectively; *p* = 0.91) and moderate exercise (3.4 ± 2.4 and 2.0 ± 1.8, respectively *p* = 0.12), but differed in frequency of self-reported strenuous physical activity (1.5 ± 1.9 and 3.8 ± 2.0, respectively; *p* < 0.01). Cancer survivors post-anthracycline-based treatment had marginally lower resting LVEF (53 ± 6 and 57 ± 7, respectively; *p* = 0.07) and exercise-associated LVEF (61 ± 9 and 67 ± 8, respectively; *p* = 0.09) compared to non-cancer comparators.

VO_2 peak_ was 22% lower in cancer survivors versus non-cancer comparators (25.7 ± 7 and 34.3 ± 10.3 ml/kg/min, respectively; *p* = 0.005). Compared to controls, cancer survivors trended toward more paraspinal IMF (13.9 ± 5.6 and 11.7 ± 4.3 cm^2^, respectively; *p* = 0.11) and a higher IMF:SM ratio (0.26 ± 0.10 and 0.22 ± 0.10, respectively; *p* = 0.13). Among all participants, VO_2 peak_ was inversely correlated with IMF:SM (*p* < 0.001), and persisted after adjustment for LVEF, CVD-comorbidities, and other depots of adipose tissue (*p* < 0.0001 to 0.007; Table 1). These inverse correlations were seen in cancer survivors and non-cancer comparators separately (r = − 0.545, *p* = 0.04; r = − 0.721, *p* = 0.004, respectively). The correlations between IMF:SM and VO_2 peak_ in both cancer survivors and non-cancer comparators persisted after adjustment for the same variables, as correlations remained above 0.50 for cancer survivors and above 0.59 for non-cancer comparators. Specifically, the correlations between IMF:SM and VO_2 peak_ remained after adjustment for resting LVEF (p = 0.04; *p* = 0.004, respectively), for exercise-associated LVEF (*p* = 0.06; *p* < 0.001, respectively), and for CVD risk factors (*p* = 0.08; *p* = 0.003, respectively). The correlations also persisted after accounting for body composition measures of BMI (p = 0.04; *p* = 0.007, respectively), intraperitoneal fat (*p* = 0.05; *p* = 0.03, respectively); and subcutaneous fat (*p* = 0.08; *p* = 0.008, respectively).

**Table 1:** Correlations of VO_2 peak_ with IMF:SM in cancer survivors and non-cancer comparators

	IMF:SM correlation with VO_2 peak_					
Adjusted for:	Overall study population (*n* = 28)		Cancer Survivors (*n* = 14)		Non-cancer comparators (*n* = 14)	
	r	*p*-value	r	*p*-value	r	*p*-value
N/A^a^	−0.67	0.0001	−0.54	0.044	−0.72	0.004
Resting LVEF %	−0.70	< 0.0001	−0.59	0.035	−0.74	0.004
Exercise-associated LVEF %	−0.71	< 0.0001	−0.54	0.055	−0.85	< 0.001
CVD risk factors	−0.64	0.0003	−0.50	0.083	−0.75	0.003
BMI	−0.67	0.001	−0.58	0.040	−0.71	0.007
SC fat	−0.61	0.007	−0.50	0.080	−0.70	0.008
IP fat	−0.60	0.001	−0.56	0.047	−0.59	0.033

^a^N/A: Correlation is not adjusted for any factors

*LVEF* left ventricular ejection fraction, *IMF* intermuscular fat, *SM* skeletal muscle, *BMI* body mass index, *SC* subcutaneous, *IP* intraperitoneal

## Discussion

To our knowledge, this is the first report that IMF:SM is correlated with reduced VO_2 peak_ in cancer survivors. This finding occurred in the context of a study showing reduced exercise capacity in cancer survivors versus matched non-cancer comparators, with marginal differences in resting and exercise-associated LVEF. In addition, we observed a marginal difference in IMF in cancer survivors versus non-cancer comparators. Taken together, our findings suggest that IMF:SM as well as potential decrements in LVEF, may contribute to reduced exercise capacity in cancer survivors. This concept of involvement of both skeletal muscle and cardiovascular factors in reduced exercise capacity is consistent with the literature in exercise intolerance of heart failure with preserved ejection fraction (HFpEF) and the literature in exercise capacity after cancer treatment [11, 12]. Our finding of a correlation between IMF:SM and exercise intolerance is strengthened by results showing that the relationship between IMF:SM and VO_2 peak_ was not attenuated by adjustment for visceral fat, CVD co-morbidities, or by resting LVEF or immediately post-exercise measurements of LVEF.

Prior studies have reported findings of an inverse correlation between IMF:SM and exercise capacity in non-cancer populations, similar to what our study observed. Notably, in 38 HFpEF patients, thigh IMF:SM was the strongest predictor of reduced VO_2peak_ of all body composition factors investigated, including subcutaneous fat, total thigh area, and thigh compartment area [5]. Our study focused on paraspinal IMF in the abdominal region because abdominal images are acquired frequently to stage a variety of cancers, and prior studies have used these images to assess cachexia [13]. In addition, the paraspinal muscles are well seen in abdominal MRI scans, along with depots of visceral and subcutaneous fat [14]. This allows one to distinguish ill effects of IMF relative to these other fat depots. Prior studies have shown that IMF has worse health impacts than subcutaneous fat potentially due to its role in impairing mitochondrial function, promoting muscle fibrosis, and competing with muscle for oxygen perfusion [4, 5].

While muscle wasting during cancer treatment has been appreciated for some time, less attention has been paid to changes in muscle composition. Two recent studies in cancer survivors have shown that muscle composition changes during chemotherapy. In 200 lung cancer patients, abdominal IMF increased by 7% after first-line chemotherapy in (*P* < 0.001) [15]. In 73 metastatic breast cancer patients treated with taxane-based chemotherapy, muscle attenuation decreased (indicating increased accumulation of IMF) during chemotherapy (*P* = 0.03) [16]. The importance of IMF in cancer survivors was shown by a 2017 study reporting that muscle attenuation was the strongest risk factor (of all body composition measures) for increased mortality (Hazard Ratio = 2.0 [1.3–3.1]) in 166 metastatic breast cancer patients [17]. Thus, prior research provides support for an accumulation of IMF during treatment and its impact on survival.

One limitation of the current study is its small sample size. We attempted to mitigate the impact of a small sample through the design of our study. Namely, we performed matching of cancer survivors to non-cancer comparators on age, gender, and BMI, factors most likely to impact VO_2 peak_. This efficient design reduced potential bias by minimizing the impact of confounding in our study sample, and provided for more precise correlation estimates. A second limitation relates to the timing of ascertainment of our exposures and outcomes as we were unable to examine changes in IMF:SM and VO_2 peak_ during cancer treatment. Future studies could assess whether IMF changes prior to, during, or after cancer treatment impact changes in VO_2 peak_. The change in IMF during cancer treatment is of particular interest due to the documented reduction in exercise capacity during this period in past studies [1, 18, 19].

Strengths of our study include its use of MRI to ascertain IMF area which allows for a direct assessment of intermuscular fat as shown in Fig. 1, whereas computed tomography (CT) scans calculate muscle attenuation which indirectly assesses IMF [20]. Another strength is the assessment of LVEF which allows for an examination of both cardiac and peripheral factors in relation to exercise capacity in cancer survivors compared to non-cancer comparators.

In summary, our findings suggest that IMF accumulation is an important contributor to reduced VO_2 peak_ among cancer survivors and are in accord with findings in other disorders associated with reduced VO_2 peak_ [5, 21]. These findings suggest that skeletal muscle abnormalities, in addition to heart dysfunction, contribute to the profound reductions in exercise capacity in cancer survivors, and thus need to be evaluated. Should these findings be replicated, this would provide insight into the pathophysiology of the severe, persistent exercise intolerance in cancer survivors and provide novel therapeutic targets.

## Abbreviations

a-VO_2_: arterio-venous oxygen
BMI: Body mass index
CPET: Cardiopulmonary exercise treadmill stress test
CVD: Cardiovascular disease
FACT-F: Functional assessment of cancer therapy- fatigue
HF: Heart failure
IMF: Intermuscular fat
L2: Second lumber vertebra
LV: Left ventricular
LVEF: Left ventricular ejection fraction
MRI: Magnetic resonance imaging
SM: Skeletal muscle
VO_2 peak_: peak exercise capacity
